# Authors' reply: One‐year outcomes for congenital diaphragmatic hernia

**DOI:** 10.1002/bjs5.50168

**Published:** 2019-05-09

**Authors:** S. Giuliani

**Affiliations:** ^1^ Department of Specialist Neonatal and Paediatric Surgery Great Ormond Street Hospital for Children Great Ormond Street, London WC1N 3JH UK

We agree that developing a core outcome set for congenital diaphragmatic hernia would be an important step forward in the field. To achieve this we will need to collaborate with parents, patients, neonatologists, paediatric surgeons, fetal medicine doctors, nurses, policy‐makers and other professionals caring for these babies.

With regard to the limitations of the study, these were presented extensively in the Discussion, but we still hope this paper will give original and accurate information to the scientific community.

